# Efficacy evaluation of retrospectively applying the Varian normal breathing predictive filter for volume definition and artifact reduction in 4D CT lung patients

**DOI:** 10.1120/jacmp.v15i3.4315

**Published:** 2014-05-08

**Authors:** Ciaran Malone, Luke Rock, Christina Skourou

**Affiliations:** ^1^ Department of Radiation Oncology St Luke's Radiation Oncology Network Dublin Ireland; ^2^ Department of Radiation Oncology University of Pittsburgh Medical Center, Beacon Hospital Dublin Ireland

**Keywords:** 4D CT, breathing irregularities, Varian RPM, normal breathing predictive filter

## Abstract

Phase‐based sorting of four‐dimensional computed tomography (4D CT) datasets is prone to image artifacts due to patient's breathing irregularities that occur during the image acquisition. The purpose of this study is to investigate the effect of the Varian normal breathing predictive filter (NBPF) as a retrospective phase‐sorting parameter in 4D CT. Ten 4D CT lung cancer datasets were obtained. The volumes of all tumors present, as well as the total lung volume, were calculated on the maximum intensity projection (MIP) images as well as each individual phase image. The NBPF was varied retrospectively within the available range, and changes in volume and image quality were recorded. The patients' breathing trace was analysed and the magnitude and location of any breathing irregularities were correlated to the behavior of the NBPF. The NBPF was found to have a considerable effect on the quality of the images in MIP and single‐phase datasets. When used appropriately, the NBPF is shown to have the ability to account for and correct image artifacts. However, when turned off (0%) or set above a critical level (approximately 40%), it resulted in erroneous volume reconstructions with variations in tumor volume up to 26.6%. Those phases associated with peak inspiration were found to be more susceptible to changes in the NBPF. The NBPF settings selected prior to exporting the breathing trace for patients evaluated using 4D CT directly affect the accuracy of the targeting and volume estimation of lung tumors. Recommendations are made to address potential errors in patient anatomy introduced by breathing irregularities, specifically deep breath or cough irregularities, by implementing the proper settings and use of this tool.

PACS numbers: 87.57.Q‐, 87.57.C‐, 87.57.N‐, 87.57.nf, 87.55.D‐.

## I. INTRODUCTION

Effective treatment of lung cancer requires an accurate representation of the anatomy for dose calculation. Precise information on tumor and organ motion is, therefore, of crucial importance.^1^, ^2^ Tumor motion is not predictable by tumor size, location or pulmonary function test results.^(3)^ Therefore, it is recommended that tumor motion is measured individually for all relevant patients.^2^, ^3^ Knowledge of this motion makes it possible to reduce the planning target volume (PTV) margins to spare normal tissue while having the possibility of dose intensification to the tumor area, which consequently maximizes the benefits of the treatment.^(4)^ Information on target motion can be acquired by a 4D CT‐enabled imaging system and is subsequently made available for target definition prior to treatment planning(2,5) It is therefore important to have a full understanding of phase sorting methods in 4D CT, especially in terms of correction techniques when dealing with breathing irregularities.

There are numerous approaches to using the information obtained from a 4D CT exam. One method is the use of the maximum intensity projection (MIP) to define the internal target volume (ITV).^6^, ^7^ The MIP image is formed using the maximum pixel value of all phases, which results in an image that represents the total extent of the tumor movement. Each phase bin represents the anatomy at a particular breathing phase. Another method is to use a single phase bin, or range of phases, usually around end expiration, for planning a radiotherapy gated treatment, as these phases represent a stable part of the patient's breathing cycle for use with respiratory gating.^8^, ^9^

The sorting of images into phase‐based volumes has been shown to be erroneous and can result in moderate to severe image artifacts.^10^, ^11^ This is caused by a lack of periodicity in patients' breathing patterns (e.g., irregularities such as coughs or sudden deep breaths). The issue of phase‐based artifacts has been discussed by Sarker et al.^(12)^ and some methods have been suggested in an attempt to reduce the presence of these artifacts ensuring the best anatomical representation of the patient from the acquired 4D CT scan.^13^, ^14^ Currently, the user can manually select, exclude, and replace individual images retrospectively in an attempt to correct image artifacts related to irregular breathing. The method described in this study makes use of technology that is readily available to every clinic using the Varian RPM System (Varian Medical Systems, Palo Alto CA).

The Varian RPM System allows the user to set a normal breathing predictive filter (NBPF) threshold in order to correct for patient's exhibiting irregular breathing. In the Varian RPM Reference Manual V1.7,^(15)^ the normal breathing predictive filter is described as a filter that “protects against misapplied radiation during treatment or automatically‐triggered prospective CT simulation.” For example, if the motion of the current breath does not match the prediction to a certain percentage, then the filter holds the beam or stops triggering CT scans. The predictive filter records a history of the pattern of amplitude versus phase values for periodic breathing by accumulating a phase‐amplitude 2D histogram. The clustering pattern of the 2D histogram bins represents the learned pattern of the breathing signal. To determine if each new sample is following the expected periodic pattern, its amplitude‐phase bin is examined for the relative level of clustering from previous samples (Varian, private communication, 2013). The degree of variation that is acceptable is determined by the threshold level set by the user. A threshold of 0% corresponds to no filtering (where all images acquired are included in the image sorting), while a threshold of 100% corresponds to the patient's breathing pattern matching the preceding trace perfectly. The default NBPF software setting is 20%. However, patients' breathing patterns and regularity cannot be predicted prior to undertaking a retrospective 4D CT simulation and, thus, limits the usefulness of setting this filter before undertaking this type of scan.

We have found that this filter can also be changed retrospectively, after 4D CT simulation, where the NBPF value chosen can filter which images are to be included and which are to be discarded in forming the patient volume. When using the filter for retrospective 4D CT acquisition, this filtering process is undertaken on the recorded breathing trace data after acquisition and does not change what data are acquired during imaging. Therefore, calling the NBPF a “predictive filter” is somewhat misleading when used for retrospective 4D CT acquisitions. Any images in the 4D CT set that are associated with a flagged region of the breathing cycle are disregarded by the system and not used in forming the patient volume. The image closest to the target phase, which has not been discarded at that slice location by the NBPF, is used instead. This allows the user freedom to apply different NBPF values to the patient's breathing trace, in order to find an appropriate filtering level, prior to exporting the data to the GE Advantage 4D software package (GE Healthcare, Waukesha, WI). The purpose of this study is to evaluate this filter as a retrospective tool, rather than its intended use as a prospective tool, and to offer recommendations on its use when dealing with common deep breath or cough irregularities.

## II. MATERIALS AND METHODS

### A. Experimental conditions

Ten 4D CT datasets were obtained from patients with tumor nodules in the lung. Each dataset was selected on the basis of having well‐defined tumors of varying size and position. Eight of the patients experienced a degree of breathing irregularity where the 4D CT reconstruction was thought likely to fail in reproducing an accurate anatomical representation, where six of these were cough or deep breath irregularities. The remaining patients had relatively regular breathing traces and were used as a reference. In regard to Patient 1, two lung nodules were identified in different locations of similar sizes, hereby referred to as I and Ia.

Patients with tumors detached from the mediastinum and lung walls were preferentially chosen. This criterion was chosen to enable the use of autocontouring methods which eliminates user‐specific contouring variability. The patients were immobilized using a vacuum bag and scanned under free‐breathing conditions using a GE Lightspeed CT simulator (GE Healthcare Systems) equipped with the Varian RPM System. The protocol used was the Advantage 4D CT cine protocol as provided by GE Healthcare, using a slice thickness of 2.5 mm. The images acquired were sorted into the default ten phase bins using the GE Advantage 4D software package, where 0% corresponds to peak inspiration and 50% end expiration.

### B. Patient breathing traces

For phase‐based sorting, the automatic phase allocation by the Varian RPM software was found to have errors in identifying the breathing peaks in all ten patients where local maxima were missed or the software chosen maxima were off‐center. These errors were manually corrected for all ten patients, using the Varian RPM Systems ‘Review’ tool, before the breathing files were exported to the GE Advantage 4D package. It is very important that each peak be properly identified, as the software uses a linear interpolation between peaks to assign phases. This suggests that this method of assigning phases assumes a perfectly regular breathing trace and is not sensitive enough to compensate for irregularities in the frequency, baseline or amplitude of a patient's breathing.

**Figure 1:**
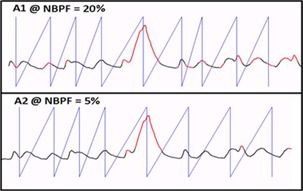
Deep breath irregularity with NBPF set at 20% (a) and 5% (b). With the NBPF reduced to 5%, a more reasonable filtering is achieved.

Patients' breathing traces were also used to evaluate the degree of the breathing irregularity experienced for each patient, where a breathing irregularity is defined as a cough or deep breath similar to breathing trace A1 in Fig. 1. Other studies have provided novel methods of quantifying the total irregularity of a breathing signal. These include a study by D. Ruan and Fessler^(16)^ who used a projection‐based model to quantify the irregularity of a signal related to respiratory motion. A second study was published by George et al.^(17)^ who employed a sinusoidal modal to evaluate the accuracy of a respiratory model for lung cancer patients. Despite of the novelty of both these studies, neither group has proposed a method of quantifying the magnitude of a single irregularity (deep breath or cough) within a breathing signal from the data contained in the breathing signal itself. Thus, we formulated an index to quantify the magnitude of each cough or deep breath irregularity to further analyze its impact. Our method consisted of exporting breathing traces and analyzing them to evaluate the regularity using the mean and standard deviation of each trace individually. Only the portion of the patient trace that was used by Advantage 4D for image sorting was evaluated. An index was formulated to compare the amplitude of one patient irregularity to another by taking the percentage amplitude deviation of the irregularity from the mean of the section of breathing trace used in the sorting of the image dataset. The index is mathematically shown below, where *x* is the mean amplitude of the breathing trace used for image sorting and *A* is the amplitude of the irregularity.

(1) $$Magnitude\;of\;Irregularity=\frac{A}{\bar{x}}\times 100$$

### C. Normal breathing predictive filter

Different NBPF thresholds were retrospectively set on the Varian RPM computer for each patient's breathing trace and exported to Advantage 4D console (GE Healthcare). NBPF thresholds used were 0%, 5%, 10%, 15%, 20%, 30%, 40%, 50%, and 60% for the default number of phase bins (10). An image dataset was deemed unfit for clinical use when the number of images flagged as invalid resulted in “gaps” in certain slice locations in the Advantage 4D software package. Using a setting over 60% commonly resulted in image sorting “gaps” (that is, too many omitted images) and therefore this value was chosen as an initial upper limit for this study. For each patient's 4D CT scan and NBPF value, the maximum intensity projection (MIP) and a reconstruction of each phase were generated. The MIP was chosen as it is a commonly used tool for reconstruction and representation of the imaging target volume (ITV) in 4D CT studies. Each individual phase was also reconstructed to evaluate the effect of the NBPF on individual phases. Any artifacts which were removed with different NBFP values were noted.

### D. Contouring

Contouring was undertaken with the GE Virtual Simulation software using Hounsfield unit ranges to autocontour the tumor, the lungs, and the patient's body. Hounsfield unit ranges were chosen that would allow the AdvantageSIM application to contour any lung tumors (approximately −150 to +600HU) and the lungs (approximately −1000 to −200HU). Tumor density between patients varied slightly and thus the HU range for each patient's tumor was selected individually, around the quoted ranges above, to ensure the tumor was fully contoured and the selected HU range for each tumor remained fixed throughout the measurements. This method eliminates any user‐specific contouring variability of over‐ or underestimating the volumes. The presence of a consistent systematic error is accepted using this method as only the relative volume changes and the absolute volumes were not used to evaluate the effect of the NBPF. The contouring was repeated for NBPF values from 0% to 60%.

### E. Volume variation

The physical gross tumor volume (GTV), lungs, and patient body volumes are unknown for this study. However, the volume associated with the default NBPF value of 0% was used as a reference volume as it allows for the comparison of having no filtering to introducing varying degrees of filtering. Relative volume deviations from this default value were recorded as percentage differences to allow for comparison between patients.

### F. Additional analysis

The RPM gating system saves a respiratory data file that can be used to evaluate the respiratory motion. The data files were exported and evaluated using a mathematical spreadsheet program to determine the proportion of images flagged in each phase bin for each patient. A number of comparisons were made with the patient's scans to evaluate useful information for use in the clinical environment. The value of the NBPF at which the tumor volume changed dramatically (>5%), hereby referred to as the “tumor volume breakdown”, was recorded and compared to the severity of the irregularity. Also, the amplitude of each deep breath or cough breathing irregularity was compared with how early the NBPF came into effect. The corresponding results, shown in Table 1 help to predict the significance of changing the NBPF with differing patient's breathing traces.

**Table 1:** Ten patients with breathing irregularities in order of magnitude including the NBPF values at tumor volume breakdown and the corresponding number of images flagged. The number of images flagged as invalid at a NBPF value of 60% is also shown

*Patient*	*Irregularity (% Difference from mean)*	*NBPF Breakdown Occurred at*:	*Images Flagged at Breakdown*	*Total Flagged Images at 60%*
9	109	60	24	24
10	114.6	60	41	41
5	118	60	92	92
2	119.5	60	26	26
6	130.7^a^	40	50	89
4	130.8^a^	40	40	125
8	153.4^a^	40	25	114
7	171.7^a^	40	26	109
3	172.7^a^	40	23	144
1	175.5^a^	30	27	172

a Denotes patients who experienced a deep breath or cough during the scan.

## III. RESULTS

### A. Patient irregularities

The severity of each patient's irregularity was evaluated using extracted data from the Varian RPM breathing trace files. The maximum irregularity deviation from the mean amplitude of the patients breathing trace ranged from 109% to 175.5%. The results of all ten patients are shown in Table 1. Deep breath and cough irregularities are highlighted for six patients. The remaining four patients experienced no significant deep breath or cough‐induced irregularity. Also shown is the NBPF level when the volume of the tumor began to dramatically change and the corresponding number of images rejected from the total number of images taken.

### B. The effect of the NBPF on the maximum intensity projection

The effect of the NBPF on the tumor volume was evaluated from 0%–60% NBPF threshold. An example of changing the NBPF filtering level and the resulting filtered portions of the patient's breathing trace are shown in Fig. 1. Images A1 and A2 of Fig. 1 show a deep breath irregularity with the NBPF set at 20% and 5%, respectively. At 20%, a significant portion of the patient's breathing trace was flagged as invalid (shown in red). When the filter was reduced to 5%, the deep breath irregularity remained filtered with far less of the remaining breathing trace flagged. Figure 1 also demonstrates the effect of a deep breath on subsequent peaks. The filter analyzes each peak by comparing it to the previously learned breathing data; thus, deep breath irregularity is included in the filters analysis, leading to the overfiltering of subsequent peaks. With the NBPF reduced to 5%, a more reasonable filtering was achieved.

**Figure 2:**
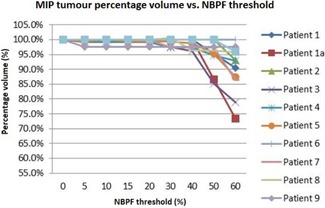
The effect of changing the NBPF on the volume of the measured tumor MIP for each of the ten patients.

It is shown in Fig. 2 that significant changes in the volume of the tumor occur as a result of the NBPF rejecting increasing numbers of images from the reconstruction of the patient volume. The change in tumor volume over ten patients ranged from 2.4% to 26.6% relative to the volume with no filtering (NBPF=0%). A NBPF above 60% was too sensitive for clinical application, as it rejected a large proportion of the images resulting in large changes in tumor volume. Changing from no filtering to 5% NBPF resulted in small changes in some patients' tumors. Above 40%, dramatic changes in tumor volume were observed for nine out of the ten patients, as images from the entire breathing trace were being removed rather than from the region of the irregularity alone. This changed the images utilized to form the entire patient volume instead of only the part of the breathing trace containing the irregularity.

The cine CT slice location where an irregularity occurs within the patient is important. It was observed that if the irregularity occurred near the tumor, the tumor volumes experienced greater changes at lower NBPF threshold values, as images associated with this location were being flagged as invalid. Thus, greater care should be taken where an irregularity occurs over a region that is clinically significant (e.g., the tumor or lung diaphragm region) for lung cancer. However, with larger NBPF values, images from the entire breathing cycle are flagged as invalid, which affects the entire patient volume.

The effect of the NBPF on the total lung volume was also evaluated from 0%‐60% threshold. Although the effect is similar to the tumor volumes, the percentage change in volume with filtering compared to no filtering for the lung was smaller than the tumor volume changes. The change in total lung volume over the ten patients was in the order of ∼1% of the volume with no filtering.

**Figure 3:**
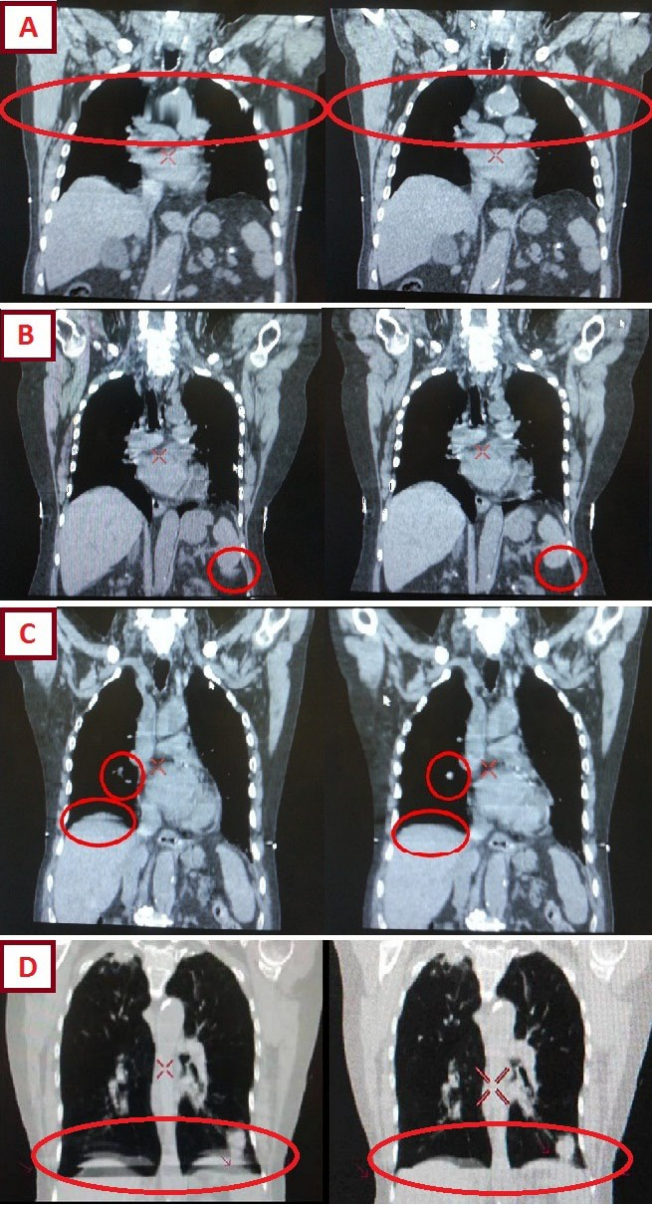
Four situations where the default filter value of 20% was not effective in correcting breathing irregularities ((a)‐(d)). Each artifact was corrected with the adjustment of the NBPF to 5%, 15%, 30%, and 20% for (a), (b), (c), and (d), respectively. An artifact found when the filter was set to 0% (d).

From these results it is clear that the NBPF can be adjusted to dramatically affect the images used in the sorting of the patient volume. From Fig. 2 it can be seen that the range of NBPF that can be used effectively is approximately 5%–40%. Figure 3 shows that a default filter value of 20% results in artifacts due to breathing irregularities. No filtering (NBPF=0%) results in incorrect image allocations, as shown in Fig. 3(d), as all images are used in the binning. Setting the NBPF to over 40% is likely to result in images from the entire breathing trace being erroneously flagged as invalid, thus resulting in a misrepresentation of the volumes of the tumor or lungs. This misrepresentation is highly dependent on the location the irregularity occurs during the scan.

### C. The effect of the NBPF on individual phases

**Figure 4:**
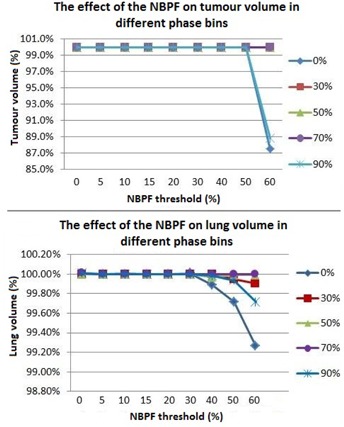
The tumor and lung volume variation with changing NBPF for each phase bin. Phases around peak inspiration were found to be greatest affected.

As the MIP represents a contribution from all phases, the 0%, 30%, 50%, 70%, and 90% phases were evaluated individually to identify if some phases are affected more than others. Phases closest to peak inspiration (0%) were found to be most affected, as the filter appears more sensitive to amplitude irregularities rather than periodic or baseline shifts. The tumor volumes had the most noticeable changes. The lung volumes exhibited changes in the order of 1%. The results for each phase bin can be seen in Fig. 4.

**Figure 5:**
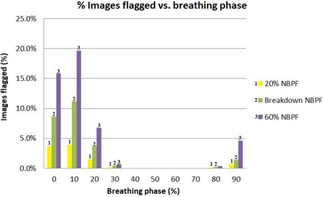
The average proportion of images flagged of all the patients for each phase bin is shown for an NBPF value of 20% at the breakdown level and at an NBPF value of 60%. The 0% and 10% phase bins show the largest effect.

Each of the patient's individual respiratory files were analyzed using a mathematical spreadsheet program to evaluate the effect of the NBPF. Figure 5 shows the average proportion of flagged data points contained in the respiratory files of all ten patients, which were flagged as invalid in each phase bin. Phases around peak inspiration (0%) contain more data points flagged as invalid than phases at end expiration (50%), with the 10% phase bin being the most affected by the filtering. Thus, when retrospectively evaluating a 4D CT scan containing an irregularity, it is recommended that the phases associated with peak inspiration be checked first for artifacts in image sorting. Figure 3 demonstrates noted artifacts as a result of irregular breathing. Each image corresponds to either the peak inspiration phase bins (3(a) and 3(d)), which were determined independently to be the most affected, as shown in Fig. 5, or the phases close to peak inspiration (3(b) and 3(c)). No artifacts were noted in the end‐expiration phase bins.

Figure 5 also shows the average proportion of images flagged at three different NBPF values where a 20% NBPF setting is the Varian RPM software's default value and the 40%‐60% NBPF values demonstrate the individual phase bin proportions where image gaps occurred, referred to as “breakdown”, for each patient. At 20% NBPF, the maximum proportion of images flagged as invalid occurred in the 10% phase bin with an average of 3.9% (SD=0.06%) of the phase bins images flagged. In comparison, the breakdown region experienced a maximum also in the 10% phase bin where an average of 11.2% (SD=0.15%) of the phase bins images were flagged. Lastly, at the maximum NBPF tested (60%), the 10% phase bin experienced a maximum where on average 19.7% (SD=0.18%) of the phase bins images were flagged. Therefore, it is recommended that phases around peak inspiration be checked for excessive flagged images, and care should be taken to ensure the patient's reconstructed volume is correct.

## IV. DISCUSSION

In this study, we investigated the effect of the Varian RPM normal breathing predictive filter on the quality of volume reconstruction and quality of image phase‐based sorting following 4D CT data acquisition. Images acquired during unusual patient behavior (such as a deep breath or a sudden cough) need to be identified and excluded from the 4D image reconstruction. The NBPF has not been previously evaluated in depth to describe its behavior and its effect on image sorting and volume definition.

Multiple studies have compared target volumes resulting from reconstructed MIP images against volumes obtained from combining the targets drawn on individual phases.^18^, ^19^ All studies conclude that the ITV defined on a MIP reconstruction is underestimating the extent of the tumor motion as defined by observing each individual phase image. James et al.^(20)^ have confirmed that ITV margins deduced from MIP reconstructed images were inadequate to provide full cover for true tumor location in any of the patients studied. All the aforementioned studies failed to include image sorting techniques as a source of error.

Cai et al.^(21)^ introduced intentional coughs to a phantom study that were undetected by the 4D CT reconstruction strategies and resulted in an error in estimating the ITV by using the MIP as a contouring tool of up to 40%. They suggest either introducing a more rigorous coaching method for the patients to ensure a reproducible breathing cycle or taking in account the potential error when defining the ITV margin from the reconstructed MIP. Our study suggests the use of an existing tool to obtain greater confidence on the reconstruction of the MIP by better managing the sorting mechanism used by 4D CT scanners.

To set the appropriate NBPF value upon completion of the scan, the RPM camera software's “Review” feature was found to be a useful tool in observing and evaluating flagged portions of the breathing trace, prior to exporting the breathing trace file to the Advantage 4D terminal. Therefore, one can evaluate the portions of the breathing trace that were deemed “irregular” by the NBPF to ensure they correspond to irregular portions of the breathing trace. An example of this process is shown in Fig. 1, and its success in identifying and removing significant irregularities such as coughs or deep breaths was demonstrated in this study, as shown in Fig. 3. The example cases in Figs. 3(a), (b), and (c) represent three situations where the default filter value of 20% was not effective in correcting breathing irregularities resulting in image artifacts. An example of an image artifact when the filter was set to 0% is also shown in Fig. 3(d). Each artifact in Fig. 3 was corrected with the adjustment of the NBPF to 5%, 15%, 30%, and 20% for images (a), (b), (c), and (d), respectively.

Our work has shown that the filter has a direct effect on the sorting of images that form the volume of the patient's structures. The minimum effect any such changes has on the volume of larger organs (such as the lung) is also demonstrated. Therefore, use of this tool can result in improved reconstruction of the tumor without compromising the depiction of other volumes of interest.

The results of this study also suggest that care should be taken in using the NBPF. Firstly, the filter should not be turned off (set to 0%) which allows the software to use all the images in reconstructing the phases. Similarly, increasing the filter to over 40% increases its sensitivity to deviations from a consistent breathing profile and results in rejection of an excessive number of images, significantly underestimating the volume of interest.

Setting the NBPF to 60% resulted in tumor volume changes of up to 26.6% of the original tumor volume, depending on the location of the breathing irregularity, whilst changes of the order of 1% are observed in the lung. It was observed that the closer the irregularity to the tumor, the more significant the effect of the NBPF on the tumor structure and volume.

The study has also shown that the filter is more sensitive to irregularities in some phases than others. Peak inspiration phases were much more likely to be affected compared to end expiration, with the 10% phase bin experiencing the largest effect. Thus when evaluating a 4D CT phase‐sorted image set, or when a new NBPF value is selected, phases around peak inspiration should be checked first for image sorting discrepancies. Also, the phases associated with end expiration showed little‐to‐no response to the changing NBPF. This suggests that the filter is responding to amplitude changes and is not sensitive to irregularities at end expiration, baseline shifts, or frequency changes.

The proportion of images flagged for each phase bin should also be monitored, especially if it approaches 10% of the total images in a particular phase bin, as it was shown to result in significant changes in the structure volumes. Rejection of large proportion of images will result in misrepresentation of the true tumor and organ volume. The user can, therefore, confidently allow a larger number of images to be rejected the flagged images are spread out evenly over multiple phase bins.

Therefore, selecting an appropriate filtering level depends both on the magnitude and type of irregularity and needs to be evaluated on a patient‐by‐patient basis. Irregularities of greater magnitude caused the filter to respond and flag images associated with the entire breathing trace at an earlier value. Thus, care should be taken in choosing a filter value, especially a value outside the range of 5%–40%.

By studying the behavior of NBPF as a retrospective tool, the potential of this filter to improve volume definition by minimizing errors in image reconstruction caused by irregular breathing in 4D CT image acquisition has been revealed. With the MIP reconstruction being the most widely used method for target delineation, minimizing potential errors resulting from breathing irregularities will reduce the risk of overestimating or underestimating the target volume. To better avoid this risk, the images from individual phases should still be used to evaluate the overall coverage of the target drawn on a MIP reconstruction.

## V. CONCLUSIONS

In this study we investigated the Varian RPM normal breathing predictive filter as a tool in addressing deep breath or cough irregularities in lung cancer patients undergoing target delineation using 4D CT. Currently the normal breathing predictive filter is used as a prospective tool. However, we have evaluated its use as a retrospective tool in managing breathing irregularities and filtering images throughout the breathing cycle that do not meet its tolerances. The NBPF's effect on both individual phases and maximum intensity projections were evaluated, and the clinically useful range was found to be in the range of 5%–40%, where either turning the filter off or setting it to >40% resulted in dramatic tumor volume changes. Also, in evaluating a 4D CT dataset for irregularities when using the filter, phases around peak inspiration should be evaluated first, as the filter is much more likely to affect these phases than mid‐ or end‐expiration phases. With informed use of this tool it is possible to automatically filter patients' breathing traces to correct for deep breath or sudden couch irregularities and thus reduce image sorting artifacts that may result in erroneous calculation of tumor volume.

## ACKNOWLEDGMENTS

The authors would like to thank Colin Kelly (St.Luke's Radiation Oncology Network, Dublin) for his guidance in the initial stages of this work.

## Supporting information

Supplementary Material

Supplementary Material

Supplementary Material
