# Supplementary figures and images for: Efficacy evaluation of retrospectively applying the Varian normal breathing predictive filter for volume definition and artifact reduction in 4D CT lung patients
Source: J Appl Clin Med Phys. 2014 May 8;15(3):14–24. doi: 10.1120/jacmp.v15i3.4315 (PMC5711067; doi:10.1120/jacmp.v15i3.4315)

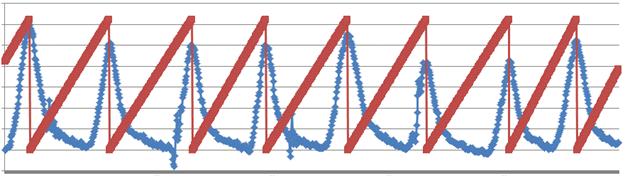

Supplement: Supplementary file 1 — Supplementary Material [file ACM2-15-014-s001.jpg]

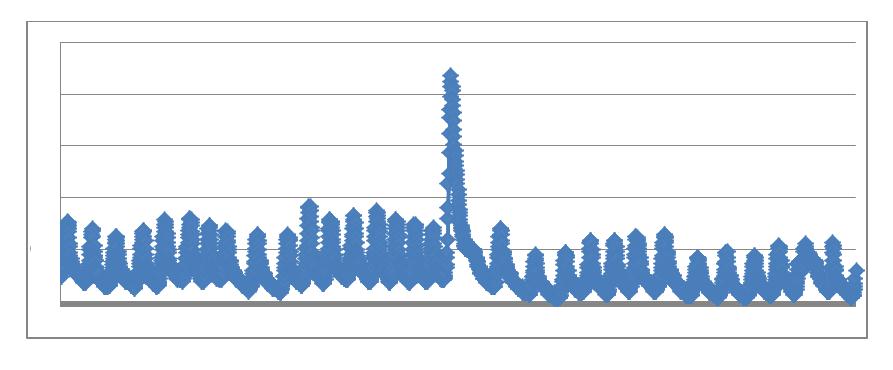

Supplement: Supplementary file 2 — Supplementary Material [file ACM2-15-014-s002.jpg]

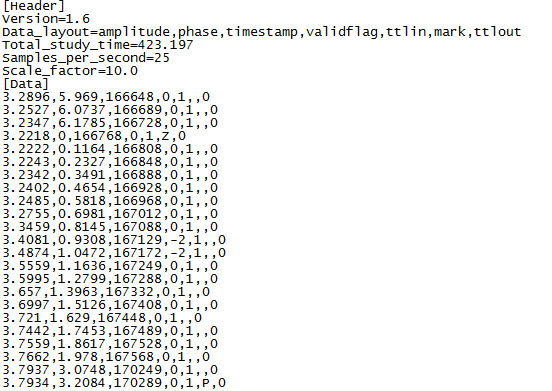

Supplement: Supplementary file 3 — Supplementary Material [file ACM2-15-014-s003.png]
